# Bicyclol induces cell cycle arrest and autophagy in HepG2 human hepatocellular carcinoma cells through the PI3K/AKT and Ras/Raf/MEK/ERK pathways

**DOI:** 10.1186/s12885-016-2767-2

**Published:** 2016-09-21

**Authors:** Yu Wang, Hao Nie, Xin Zhao, Yong Qin, Xingguo Gong

**Affiliations:** Institute of Biochemistry, College of Life Sciences, Zijingang campus, Zhejiang University, Room 345, Hangzhou, 310058 Zhejiang China

**Keywords:** Bicyclol, Cell cycle, Autophagy, AKT, ERK, HepG2

## Abstract

**Background:**

Bicyclol, a novel synthetic antihepatitis drug, is widely known to protect against liver injury. However, few reports have focused on the possible effect of bicyclol on anti-proliferation and autophagy induction in cancer cells, particularly hepatocellular carcinoma cells.

**Methods:**

In this study, we investigated the antitumor efficacy of Bicyclol in HepG2 cells and the mechanism of cell growth inhibition. Cell proliferation was analyzed by MTT assay, and the cell cycle and apoptosis were assessed by flow cytometry. And we transfected the cells with the GFP-RFP-LC3 vector to detect the autophagy flux in the cells. Mechanisms of bicyclol-induced cell growth inhibition were probed by western blot analysis.

**Results:**

Bicyclol effectively inhibited HepG2 cell proliferation in a dose- and time-dependent manner. In addition, we found that bicyclol inhibited cell cycle progression at G1 phase and induced autophagy in HepG2 cells, which implied that the significant decrease in cell proliferation was mainly induced by autophagy and inhibition of cell proliferation. Furthermore, western blot showed that bicyclol inhibited phosphorylation of Akt and ERK, down-regulated the expressions of cyclin D1, cyclin E2, CDK2, CDK4, p-Rb and p-mTOR. Moreover, AKT or ERK knockdown by siRNA enhanced bicyclol-induced autophagy and inhibition of cell proliferation.

**Conclusion:**

These results suggest that bicyclol has potent anti-proliferative activity against malignant human hepatoma cells via modulation of the PI3K/AKT pathway and the Ras/Raf/MEK/ERK pathway, and indicate that bicyclol is a potential liver cancer drug worthy of further research and development.

**Electronic supplementary material:**

The online version of this article (doi:10.1186/s12885-016-2767-2) contains supplementary material, which is available to authorized users.

## Background

Liver cancer is the fifth most common cancer worldwide, and the second most frequent cause of cancer death [1]. The highest liver cancer rates and deaths were found to occur in China in 2008 [2]. In the USA, the liver cancer incidence rates continued to increase by at least 3 % per year from 1992 to 2009, which was the highest of all cancers. Despite extensive research into treatments of liver cancer, such as chemotherapy, hepatectomy, liver transplantation, microspheres, and immunotherapy, survival rates are 3–5 % in cancer registries in developed countries, and consistently low rates are estimated worldwide [3, 4], which highlights the urgent need for novel effective therapeutic approaches.

**Fig. 1:**
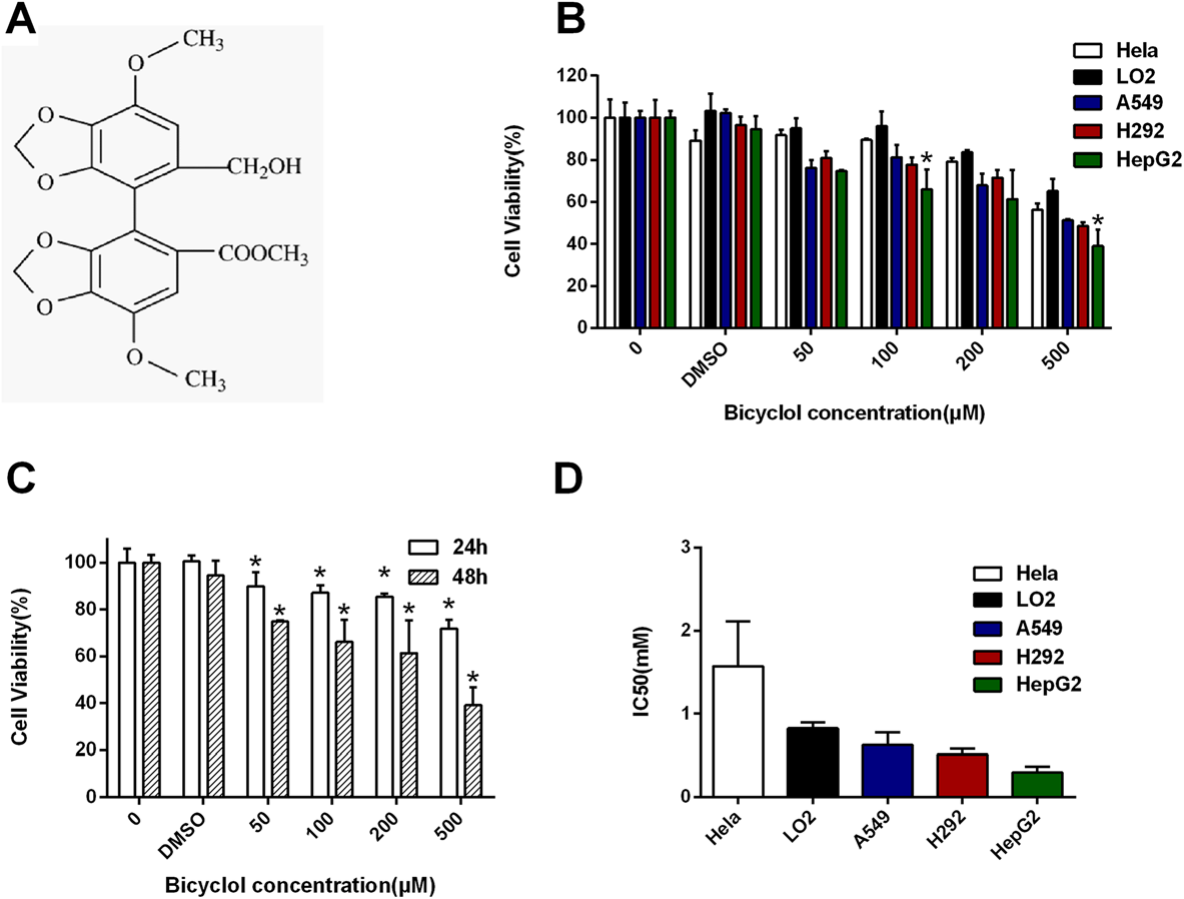
The effect of bicyclol on the living cell number of cancer cell lines and normal liver cells. **a** Chemical structure of bicyclol. **b** The effect of various concentrations of bicyclol on HepG2, Hela and LO2 cells after 48 h of treatment. DMSO-treated (0.25 %) cells were used as vehicle controls. A570 was measured after the MTT incubation. **c** dose- and time-dependent effect of bicyclol on the living cell number of HepG2 cells. The bar graphs represent the means ± SD from three independent experiments. **d** The IC50 values at 48 h in different cells. The bar graphs represent the 95 % confidence intervals

Bicyclol (4,4’-dimethoxy-5,6,5’,6’-Bis(dimethylene-dioxy)-2-hydroxymethyl-2’-methoxy carbonyl biphenyl, Fig. 1a [5]) is a new synthetic antihepatitis drug. It has been widely used in the clinic to treat patients with chronic hepatitis B viral infections [6]. In mice and rats, bicyclol effectively protects against liver injury induced by various hepatotoxins, such as acetaminophen [7], CCl_4_ [8], alcohol [9], concanavalin A [6], lipopolysaccharide and d-galactosamine [5]. Additionally, bicyclol can improve liver function and partially inhibits hepatitis B virus replication in the clinic [10]. Recently, it was reported that bicyclol effectively induces the cytoprotective effect of heat shock protein 27/70 by suppressing NF-kB in mice [11, 12], and it has similar effects in HepG2 cells through the mitochondria-associated pathway [13]. However, there were few studies about the possible effect of bicyclol on anti-proliferation and autophagy induction in cancer cells, particularly hepatocellular carcinoma cells.

Recent studies have shown that a series of chemical compounds have anti-proliferation effect in cancer cells through the PI3K/AKT pathway. The PI3K/Akt pathway plays an important role in angiogenesis, apoptosis, cell cycle progression, cell survival and cell differentiation. Upon PI3K activation, the Akt PH domain interacts with PtdIns(3,4,5)P3 and recruits Akt to the plasma membrane, where it is then activated through phosphorylation at Thr308 in the activation loop of the catalytic domain and Ser473 in the regulatory domain [14, 15]. Akt modulates the function of many downstream substrates, such as mTOR, p27 and Mdm2, which are involved in the regulation of the cellular processes mentioned above [16].

In hepatocellular carcinoma cells (HCC), the PI3K/Akt/mTOR pathway and the Ras/Raf/MEK/ERK pathway have a synergetic relationship in regulating the proliferation of tumor cells [17]. The classic Ras/Raf/MEK/ERK pathway is a key signal transduction component of cell proliferation in many cells [18]. It contains a cascade of protein kinases: Ras, Raf, MEK, and ERK. One of the key roles of the Ras/Raf/MEK/ERK pathway in many cell types is the regulation of the cell division cycle [19]. It is reported that the p27Kip1 expression is induced by Ras/Raf/MEK/ERK pathway inhibition, and cyclin/cyclin-dependent kinase 2 (CDK2) activity was also inhibited [20].

In the present study, we investigated the effects of bicyclol on HepG2 cells and further examined the cell anti-proliferation mechanism. Our observations demonstrate that bicyclol effectively inhibits HepG2 cell proliferation, but is minimally toxic to normal liver LO2 cells; the significant decrease in cell proliferation was mainly induced by autophagy and inhibition of cell proliferation. Mechanistically, we further identified the cytotoxicity of bicyclol is closely associated with the inhibition of the PI3K/AKT and Ras/Raf/MEK/ERK pathways. These preclinical studies suggest that bicyclol could be useful for the treatment of liver cancer.

## Methods

### Materials

Bicyclol (≥98 %, HPLC) was purchased from Sigma, dissolved in dimethylsulfoxide (DMSO) and diluted to the desired concentration before use; the final concentration of DMSO was less than 0.3 % in culture. 3-(4,5-Dimethylthiazol-2-yl)-2,5-diphenyltetrazolium bromide (MTT) was purchased from Sigma.

The primary antibodies were purchased from the following companies: Cell Signaling Technology (p-Rb, Cyclin D1, Cyclin E2, LC3, p-mTOR) and Sangon Biotech (p21, p27, CDK2, CDK4, Akt, p-Akt, p-ERK, Ras). All other chemical reagents were of the highest purity available. The secondary antibody conjugated with Alexa Fluor® 680 was purchased from Jackson ImmunoResearch Laboratories, Inc. The cell cycle and apoptosis analysis kit was purchased from Beyotime Biotechnology.

The LO2 (human normal liver), HepG2 (hepatocellular adenocarcinoma), A549 (human lung epithelial cells), H292 (human mucoepidermoid pulmonary carcinoma) and HeLa (cervical carcinoma) cell lines were obtained from the Cell Bank of Type Culture Collection of Chinese Academy of Sciences (Shanghai, China). All cell lines were cultured in RPMI 1640 medium (Gibco), which contained 10 % (v/v) fetal calf serum (Gibco), 100 units/ml penicillin, and 100 units/ml streptomycin. The cell lines were cultured in a humidified cell incubator at 37 °C with a 5 % CO_2_ atmosphere.

LY294002, 3-MA, benzyloxycarbonyl-Val-Ala-Asp-(OMe) fluoromethyl ketone (Z-VAD) and PD98059 were purchased from Beyotime Biotechnology. The AKT1-cDNA-pCMV expression vector was purchased from Sino Biological Inc. The AKT1 and ERK1 siRNAs were purchased from Shanghai GenePharma Co., Ltd. The Lipofectamine 2000 and Lipofectamine RNAiMAX transfection reagents were purchased from Life Technologies.

### Cytotoxicity assay

The cell metabolism rate of the cell lines was measured using the MTT assay. Exponentially growing cells were treated for 24 h or 48 h with various concentrations (0–500 μmol/L) of bicyclol in 96-well plates. DMSO-treated cells (0.25 %) were used as vehicle controls. MTT was then added to each well, and the cells were incubated for 4 h at 37 °C in the dark. The Formazan crystals that formed were dissolved with 150 μl of DMSO. The absorbance at 570 nm was measured using a Model ELX800 microplate reader (Bio-Tek Instruments). Each test was repeated at least three times. The cell metabolism rate was calculated by the following formula: %cell metabolism rate = (mean absorbance in test wells)/(mean absorbance in control well) x 100 %.

### Cell death analysis

Cell death, including apoptosis and necrosis, was assessed by staining with an annexin V–FITC/PI kit (Sigma), according to the manufacturer’s instructions. Briefly, the cells were cultured with various concentrations of bicyclol for 48 h, and then 1 × 10^6^ cells were harvested and washed twice with ice-cold PBS. The apoptotic (Annexin V+/PI-) or necrotic cells (Annexin V+/PI+) were evaluated by double staining with annexin V–FITC and PI in binding buffer using flow cytometry (FAC sort, Becton Dickinson).

### Cell cycle analysis

The cell cycle was analyzed by flow cytometry (FAC sort, Becton Dickinson). The cells were cultured with various concentrations of bicyclol for 24 h, or with 200 μl of bicyclol for 8,16 or 24 h, and then suspended in 70 % ethanol and fixed overnight at 4 °C. The cells were then treated with 20 μg/ml RNase A, followed by 25 μg/ml propidium iodide (PI). The proportion of cells in G0/G1, S and G2/M phases were determined by examining the intensity of PI fluorescence with a flow cytometer using an argon laser and 570 nm bandpass filters.

### Transient transfection and immunofluorescence

The GFP-RFP-LC3 expression vector is widely used to detect autophagic flux [21]. The cells were transiently transfected with the GFP-RFP-LC3 expression vector (kindly provided by Prof. Mao Xiang [22]) using Lipofectamine 2000, according to the manufacturer’s instructions. After the GFP-RFP-LC3-transfected cells were incubated for 48 h, the cells were treated with bicyclol for an additional 24 h. The GFP-RFP-LC3 fluorescence was observed using an Olympus FV1000 confocal microscope, and the autophagosomes (yellow dots) and autolysosomes (free red dots) in each cell were counted.

### Transient transfection of the activated AKT cDNA

The HepG2 cells were transiently transfected with an AKT1-cDNA-pCMV expression vector using Lipofectamine 2000, according to the manufacturer’s instructions as described above. After the AKT-cDNA–transfected cells were incubated for 48 h, the cells were treated with bicyclol for an additional 24 h. The subsequent assays were analyzed.

### Chemical inhibition

The HepG2 cells were cultured and pre-treated with 20 μM PD98059 for 30 min, and then the cells were treated with various concentrations of bicyclol. Bicyclol and 10 μM LY294002 were added to the cells at the same time. The subsequent assays were analyzed.

### siRNA knockdown of AKT and ERK1 expression

The HepG2 cells were transiently transfected with AKT1 siRNA duplexes (sense, GGGCACUUUCGGCAAGGUGtt; antisense, CACCUUGCCGAAAGUGCCCtt) [23], ERK1 siRNA duplexes (sense, GAGCCGCCGCCGCCGCCATtt; antisense, ATGGCGGCGGCGGCGGCTCtt) [24], or non-specific control siRNA duplexes (GenePharma Co, Ltd) using the Lipofectamine RNAiMAX reagent, according to the manufacturer’s instructions. After the siRNA–transfected cells were incubated for 48 h, the cells were treated with bicyclol for an additional 24 h. The subsequent assays were analyzed.

### Western blot analysis

The treated cells were collected, washed in PBS and then lysed with lysis buffer on ice. Approximately 20 μg of the lysed proteins were separated by sodium dodecyl sulfate-PAGE and transferred to a nitrocellulose blotting membrane. The membranes were blocked overnight in blocking buffer (5 % bovine serum albumin solution and 0.05 % Tween 20 in Tris-buffered saline (TBST)). After three washes in TBST, the membranes were probed with the indicated primary antibodies in blocking buffer for 1 h. After three washes in TBST, the blots were incubated with the appropriate secondary antibodies for 1 h in blocking buffer. After three washes in TBST for 15 min, the proteins were visualized by an Odyssey Imager (LI-COR).

### Transmission electron microscopy (TEM)

After 24 h of bicyclol treatment, the cells were collected and then fixed in 2.5 % glutaraldehyde in phosphate buffer (0.1 M, pH7.0) overnight. After three washes, the specimen was fixed with 1 % OsO_4_ in phosphate buffer (0.1 M, pH7.0) for 1 h. After washing, the specimen was first dehydrated by a graded ethanol series (30, 50, 70, 80, 90 and 100 %) for approximately 15 min at each step, and then incubated in pure acetone for 20 min. Then, the specimen was placed in a 1:1 mixture of pure acetone and the final resin mixture for 1 h, a 1:3 mixture of pure acetone and the final resin mixture for 3 h, and the final spur resin mixture overnight. The specimen was then placed in spur resin and heated at 70 °C for more than 9 h. Finally, the specimen was sectioned on a LEICA EM UC7 ultramicrotome, and sections were stained by uranyl acetate and alkaline lead citrate for 5 min, respectively. The ultra-thin sections were viewed on a Hitachi Model H-7650 TEM.

### Statistical analysis

The experimental results are expressed as the means ± SD. The changes in the different assays were analyzed by the analysis of variance followed by Student’s *t* test. A value of *P* < 0.05 was considered to be statistically significant.

## Results

### Bicyclol induced cell anti-proliferation, but not apoptosis

To examine whether bicyclol induces cytotoxic effects on different types of cancer cells, we treated HepG2, Hela, H292, A549 and LO2 cells with different concentrations of Bicyclol (0, 50, 100, 200 and 500 μM) for 48 h. DMSO-treated (0.25 %) cells were used as a vehicle control (Fig. 1b). After a 48 h exposure in 500 μM bicyclol, the living cell number of HepG2 cells was significantly reduced to 39.1 %. Meanwhile, the inhibitory effect of bicyclol on Hela, LO2, A549 and H292 cells was less than the HepG2 cells. Bicyclol inhibited HepG2 cell proliferation in a time- and dose-dependent manner (Fig. 1c). These results indicated that bicyclol had different effects on hepatocellular carcinoma from normal liver cells and other tumor cells. The IC_50_ value for bicyclol in HepG2 cells is 0.30 mM after a 48 h treatment (Fig. 1d).

**Fig. 2:**
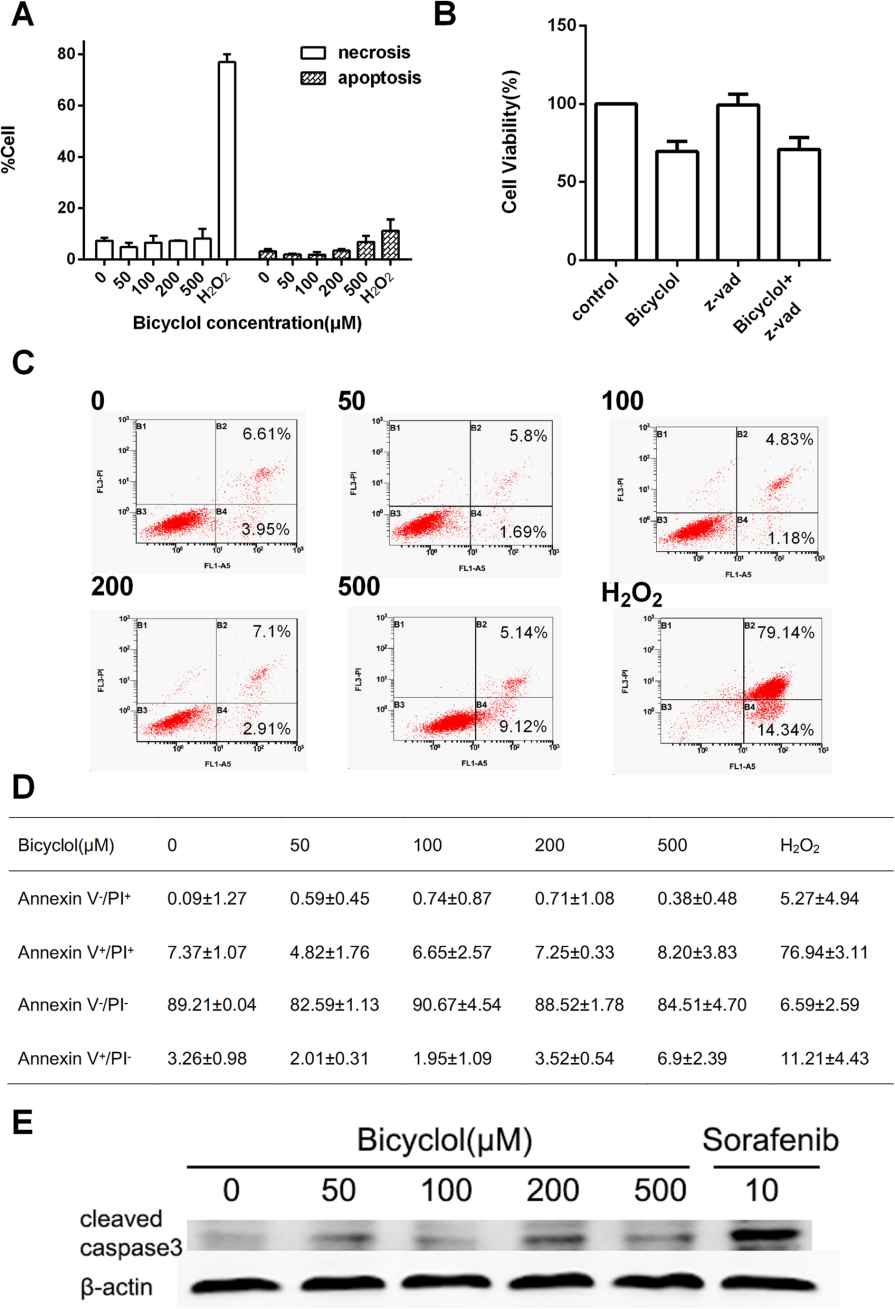
Bicyclol did not induce apoptosis or necrosis in HepG2 cells. **a** The percent of apoptotic and the necrotic cells after 24 h of treatment with different concentrations of bicyclol were measured by flow cytometry. H_2_O_2_-treated (10 μM) cells were used as positive controls. **b** Living cell number after co- treatment with bicyclol and z-vad. HepG2 cells were treated with 20 μM z-vad and 500 μM bicyclol at the same time. The cells treated with either 20 μM z-vad or 200 μM bicyclol were used as controls. After a 24 h exposure, the cells were incubated with MTT and the A_570_ was measured. **c** Flow cytometry analysis of cancer cell apoptosis using the Annexin V-FITC/PI dual-labeling technique. The B2 gate (Annexin V^+^/PI^+^) represents the percentage of necrotic cells, while the B4 gate (Annexin V^+^/PI^−^) represents the percentage of apoptotic cells. Up to 10,000 cells were counted in each sample. **d** The percent of cells identified by flow cytometry. **e** The protein level of cleaved caspase-3 treated by bicyclol and Sorafenib

We next investigated whether apoptosis could be the cause of the bicyclol-induced cell anti-proliferation; thus, an Annexin V-FITC/PI double staining assay was performed. The apoptotic (Annexin V^+^/PI^−^) or necrotic cells (Annexin V^+^/PI^+^) were identified by flow cytometry (Fig. 2). As shown in Fig. 2a, c, d, no significant increase in the number of necrotic cells was detected at any concentration of bicyclol used in this study, particularly compared with the positive control, 10 μM H_2_O_2_. Only 500 μM bicyclol slightly increased the number of apoptotic cells, but the results were not statistically significant. Furthermore, we treated HepG2 cells with both bicyclol and the pan-caspase inhibitor Z-VAD, which blocks cell apoptosis. As shown in Fig. 2b, the cell proliferation after the co-treatment was similar to the treatment with bicyclol only. And the protein level of cleaved caspase-3 was investigated. As shown in Fig. 2e, no significant increase in the protein level of cleaved caspase-3, an apoptosis indicator, was detected at any concentration of bicyclol used, particularly compared with the positive control, 10 μM Sorafenib, while Sorafenib effectively reduced cell viability (Additional file 1B) These results indicated that the bicyclol-induced cell anti-proliferation was not dependent on apoptosis.

### Bicyclol induced cell cycle arrest and suppressed the growth regulatory signals in G1 phase

**Fig. 3:**
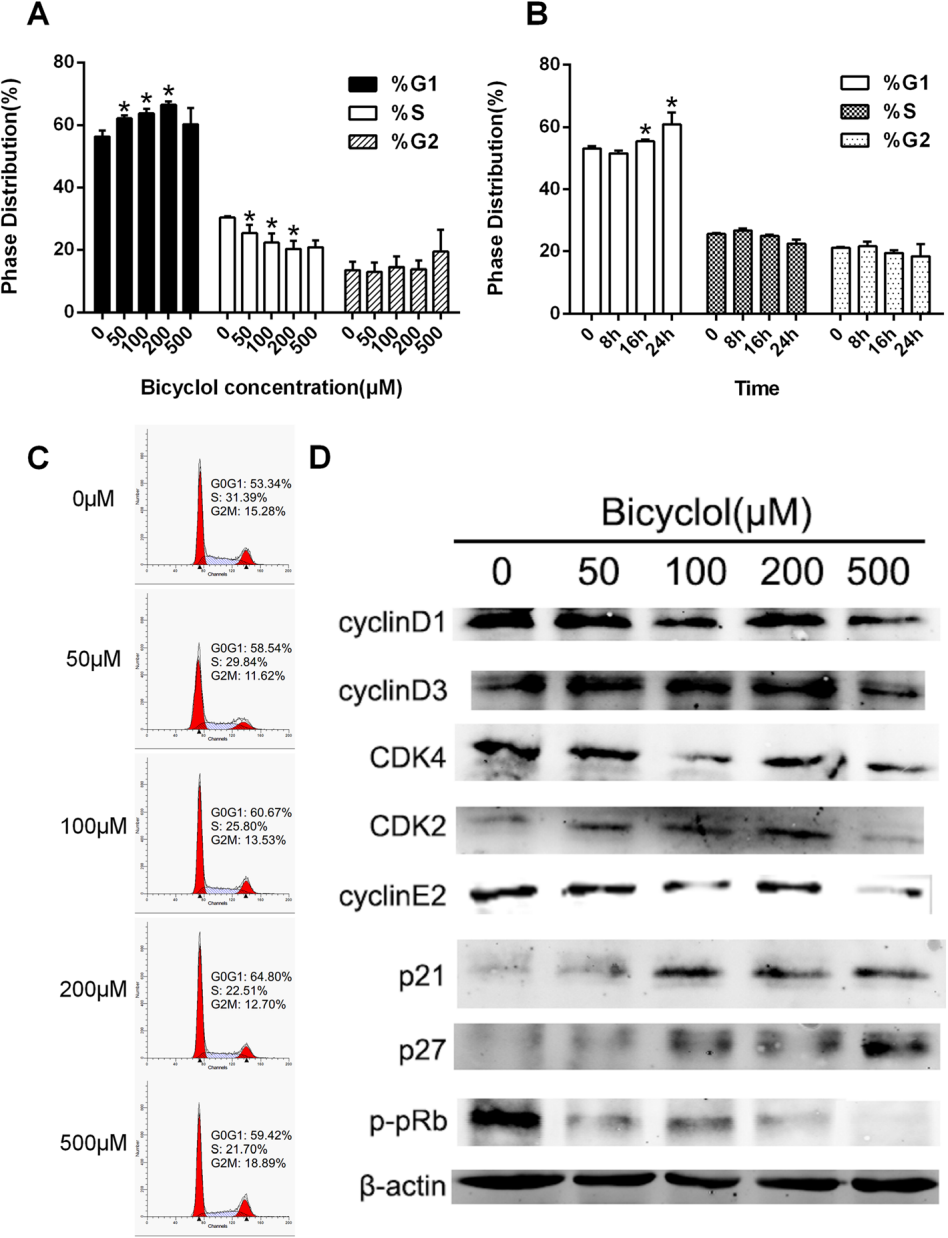
Bicyclol induced G1 cycle arrest in HepG2 cells on a dose- and time-dependent manner. **a** The phase distribution of HepG2 cells treated with various concentrations (0, 50, 100, 200 and 500 μM) of bicyclol for 24 h was analyzed by flow cytometry. The HepG2 cells were plated in six-well plates and cultured until they reached 60 % confluence. The cells were incubated in serum-free RPMI 1640 culture medium for 24 h to be synchronized. Then the cells were treated with bicyclol for 24 h. The cell cycle distribution was determined by flow cytometry with the propidium iodide (PI) dye, and distribution of cells in G1, S, and G2 phases was calculated using the Cell Quest software. **b** The phase distribution of HepG2 cells treated with 200 μM bicyclol for 8, 16 and 24 h was analyzed by flow cytometry. The cells were treated as in (A). **c** The DNA distribution of cells treated with various concentrations of bicyclol for 24 h. **d** Dose-dependent effects of bicyclol on cell cycle-related proteins in HepG2 cells. The cells were disrupted after treatment with various concentrations (0, 50, 100, 200 and 500 μM) of bicyclol for 12 h. The proteins were collected, and cellular β-actin, cyclin D1, cyclin D3, cyclin E2, CDK2, CDK4, p21, p27 and p-Rb (Ser 807) were analyzed by western blotting. (*p < 0.05 versus PBS control)

A cell cycle analysis was performed to determine how bicyclol inhibited the growth of HepG2 cells (Fig. 3). The results showed a time- and dose-dependent increase in the percentage of cells in G1 phase and a decrease of the percentage of cells in S phase after bicyclol treatment (Fig. 3a, b). 53.34 % of the PBS-treated cells were in G1 phase. After 24 h of treatment with 50, 100 and 200 μM bicyclol, the percentage of cells in G1 phase increased to 58.54, 60.67 and 64.80 %, respectively (Fig. 3c).

The growth regulatory signals of G1 phase, including Rb, cyclins, cyclin-dependent kinases and cyclin-dependent kinase inhibitors, can be further evidence of the G1/S cell cycle arrest. Phosphorylated Rb leads to the release of the E2F1 transcription factor and subsequent initiation of cell cycle progression to S phase [25]. Therefore, we next investigated the cell cycle-related protein levels in cells treated with various concentration of bicyclol using western blot (Fig. 3d). As shown in Fig. 3d, the level of phosphorylated Rb was dramatically decreased after bicyclol treatment. In addition, the expression of the cyclin D1, cyclin D3 and cyclin E2 proteins were decreased after treatment with 500 μM bicyclol. Meanwhile, the expression of the CDK2 and CDK4 proteins were decreased, while the cyclin-dependent kinase inhibitors p21^CIP^ and p27^KIP1^ were increased in a dose-dependent manner. The increase in the expression of the p21^CIP^ and p27^KIP1^ proteins and the decrease in cyclins and cyclin-dependent kinases dephosphorylate Rb and lead to cell cycle arrest, which may contribute to the anti-proliferative effects of bicyclol in HepG2 cells.

### Bicyclol induced autophagy in HepG2 cells

**Fig. 4:**
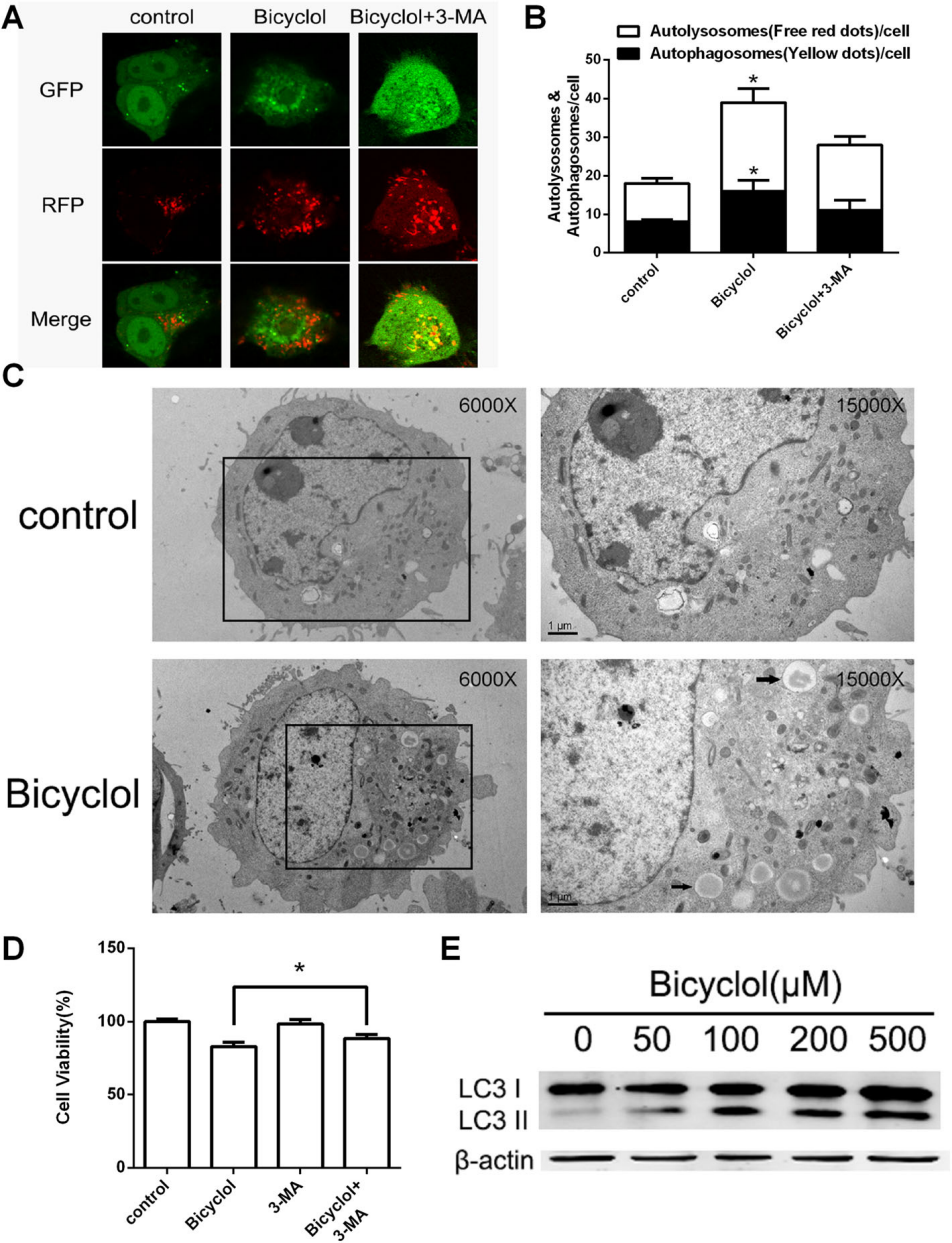
Bicyclol induced autophagy in HepG2 cells. **a** Autophagy flux was induced by bicyclol and inhibited by 3-MA. The cells were transiently transfected with GFP-RFP-LC3 vectors using Lipofectamine 2000 and incubated for 48 h, and then treated with 200 μM bicyclol for another 24 h or pre-treated with 5 mM 3-MA for 30 min. The GFP-RFP-LC3 fluorescence was observed by a confocal microscope, and the number of autophagosomes (*yellow dots*) and autolysosomes (free *red dots*) in each cell were counted by ImageJ. 50 cells for each condition were counted. **b** The number of autophagosomes and autolysosomes were increased by bicyclol, and 3-MA suppressed the effect. **c** The cellular ultrastructure was analyzed by transmission electron microscopy. The cells were incubated in 6-well plates and treated with 200 μM bicyclol for 24 h. Then, the cells were collected and fixed. Next, ultra-thin sections were viewed on a TEM. The autolysosomes were indicated by arrows. **d** Cell proliferation after co-treatment with bicyclol and 3-MA. The cells were incubated in 96-well plates and then pre-treated with 5 mM 3-MA for 30 min. Next, the 3-MA was removed and the cells were treated with 200 μM bicyclol for 24 h. The A_570_ was then measured after the MTT incubation. **e** The levels of the LC3 I and II proteins were influenced by bicyclol. The cells were treated with various concentrations of bicyclol for 12 h and then disrupted. The proteins were collected, and cellular LC3 I and II were analyzed by western blotting. β-actin was used as the loading control. Bar graphs represent the means ± SD from three independent experiments. (*p < 0.05 versus bicyclol treatment)

Autophagy is a physiological cellular strategy and survival mechanism under stress conditions. Moreover, over-activated autophagy may result in cell anti-proliferation [26]. LC3 is a hallmark of autophagy, and the conversion of cytosolic LC3-I to autophagosome membrane-bound LC3-II is a specific marker for autophagosome formation [27]. Thus, a GFP-RFP-LC3 plasmid was transfected into HepG2 cells and investigated by fluorescence microscopy. As shown in Fig. 4a, b, the amount of free red dots (indicating autolysosomes) and the amount of yellow dots (indicating autophagosomes) were significantly increased after treatment with 200 μM bicyclol. Furthermore, co-treatment with bicyclol and 3-methyladenine (3-MA, a chemical inhibitor of autophagy) reduced the autophagy-inducing and anti-proliferation effects of bicyclol (Fig. 4d). The cellular ultrastructure was analyzed by transmission electron microscopy, which markedly demonstrated the presence of bicyclol-induced autolysosomes (Fig. 4c). A western blot assay was performed to detect the conversion of LC3-I to LC3-II. As shown in Fig. 4e, the conversion was up-regulated by bicyclol in a dose-dependent manner. The results suggested that bicyclol induced autophagy in HepG2 cells.

### Bicyclol inhibited the PI3K/Akt/mTOR and the Ras/Raf/MEK/ERK pathways

**Fig. 5:**
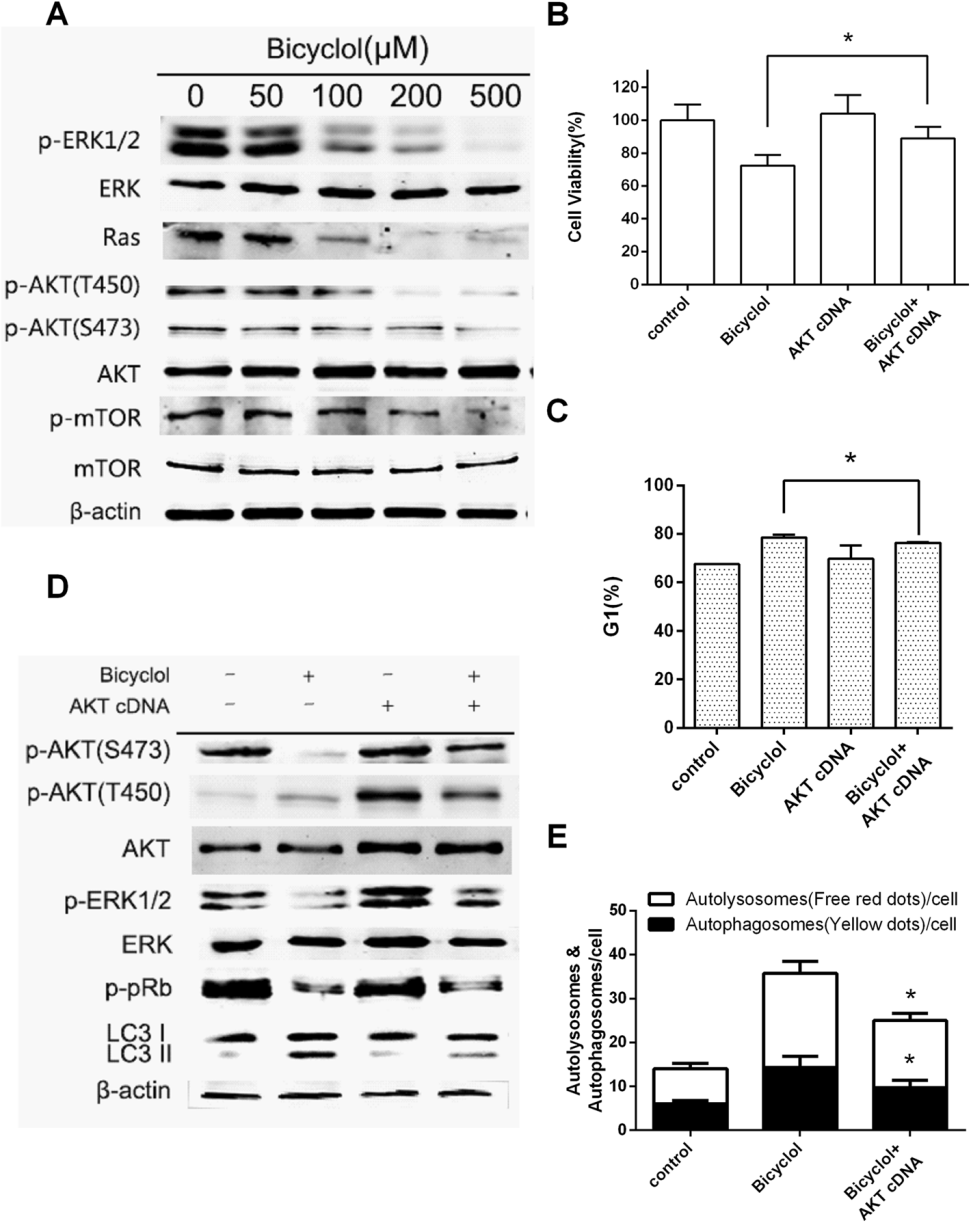
Bicyclol suppressed the PI3K/AKT and the Ras/Raf/MEK/ERK pathways. **a** Dose-dependent effects of bicyclol on the PI3K/AKT and the Ras/Raf/MEK/ERK pathway-related proteins. The cells were disrupted after treatment with various concentrations (0, 50, 100, 200 and 500 μM) of bicyclol for 6 h. The proteins were collected, and cellular β-actin, p-ERK1/2 (Thr202 and Tyr 204), total ERK, Ras, p-AKT (Thr 450), p-AKT (Ser 473), total AKT, p-mTOR (Ser 2448), and total mTOR were analyzed by western blotting. **b** The AKT cDNA rescued HepG2 cells from bicyclol-induced cell anti-proliferation. The cells were transiently transfected with the AKT cDNA expression vector using Lipofectamine 2000 and incubated for 48 h. Then, the transfected cells were treated with 200 μM bicyclol for 48 h. The A_570_ was measured after the MTT incubation. **c** The bicyclol-induced G1 arrest was reduced by the AKT cDNA. The cells were transfected with the AKT cDNA expression vector and then treated with 200 μM bicyclol for 24 h. The phase distribution was determined by flow cytometry. **d** The AKT cDNA suppressed the effect of bicyclol on the PI3K/AKT and the Ras/Raf/MEK/ERK pathways. The cells were transfected with the AKT cDNA expression vector and then treated with 200 μM bicyclol for 6 h. The cells were disrupted, and cellular β-actin, p-AKT (Thr 450), p-AKT (Ser 473), total AKT, p-ERK1/2 (Thr202 and Tyr 204), total ERK, p-Rb (Ser 807) and LC3 I and II were analyzed by western blotting. **e** The number of autophagosomes and autolysosomes were reduced by the AKT cDNA. Bar graphs represent the means ± SD from three independent experiments. (*p < 0.05 versus bicyclol treatment)

As mentioned above, bicyclol induced cell cycle arrest and autophagy in HepG2 cells. However, the pathways downstream of these bicyclol-mediated effects were investigated in-depth. As shown in Fig. 5a, Akt phosphorylation at Ser473 and Thr450 was remarkably inhibited, while the total protein level of Akt remained constant, which suggested that the PI3K/AKT pathway was involved in bicyclol-mediated cell anti-proliferation in HepG2 cells. Dephosphorylated AKT directly inhibits TSC1/2 and activates PRAS40 to inactivate mTORC1 and induce autophagy [28, 29]. Furthermore, mTOR phosphorylation at Ser2448 was inhibited after bicyclol treatment, which indicated that bicyclol induced autophagy in HepG2 cells through the PI3K/AKT/mTOR pathway.

We next investigated whether the Ras/Raf/MEK/ERK pathway was involved in the bicyclol-induced cell anti-proliferation as well. The Ras protein level was significantly reduced after bicyclol exposure. Additionally, ERK1/2 phosphorylation at Thr202 and Tyr 204 was inhibited, while the total protein level was constant. These results suggested that the synergy between the PI3K/AKT pathway and the Ras/Raf/MEK/ERK pathway played an important role in the bicyclol-induced anti-proliferative effect.

### Transfection of the constitutively active AKT cDNA suppressed the bicyclol-induced anti-proliferative effects in HepG2 cells

Our results showed that bicyclol targets the AKT signaling pathway. To confirm the role of the AKT signaling pathway in bicyclol-induced cell cycle arrest and autophagy, we next transfected HepG2 cells with a constitutively active form of the AKT cDNA and treated the AKT-overexpressing cells with bicyclol (Fig. 5b, cd, e). The expression level of total AKT was significantly increased, which confirmed the success of transfection. Transfected cells expressing the active AKT cDNA were considerably more resistant to bicyclol than cells transfected with the control cDNA. The living cell number was increased from 72.3 to 89.1 % (Fig. 5b). The bicyclol-induced cell cycle arrest was rescued after transfection, while the percentage of cells in G1 phase was decreased from 78.5 to 76.3 % (Fig. 5c, and DNA distribution was presented in Additional file 2A). Moreover, the fluorescence microscopy results showed that the amount of autolysosomes and autophagosomes were significantly decreased after transfection (Fig. 5e). Furthermore, the LC3-I to LC3-II conversion was restored in AKT-overexpressing cells compared to the control. In addition, AKT phosphorylation at Ser473 and ERK1/2 phosphorylation at Thr202 and Tyr 204 were rescued after transfection, which led to Rb phosphorylation and resulted in a decrease in the percent of cells in G1 phase (Fig. 5d). These results further confirmed that the AKT signaling pathway is indeed the target of bicyclol treatment.

### LY294002 and PD98059 enhanced the anti-proliferative effect of bicyclol

**Fig. 6:**
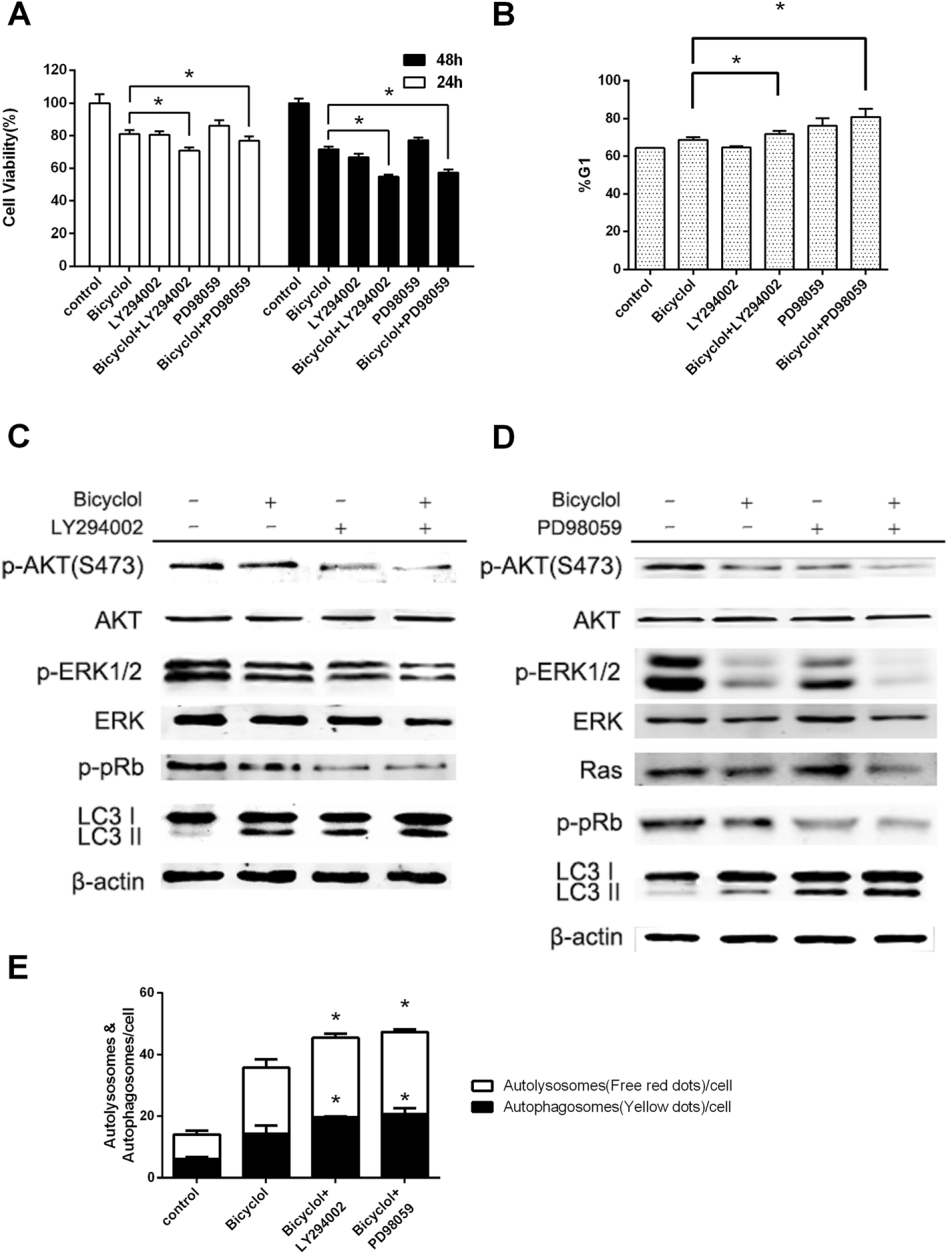
LY294002 and PD98059 enhanced the anti-proliferative effect of bicyclol in HepG2 cells. **a** Cell proliferation after co-treatment with LY294002 and bicyclol or PD98059 and bicyclol. The cells were treated with both 10 μM LY294002 and 200 μM bicyclol for 24 or 48 h, or cells were pre-treated with 20 μM PD98059 for 30 min and then 200 μM bicyclol was added to the media for 24 or 48 h. The A_570_ was then measured after the MTT incubation. **b** The percentage of cells in G1 phase after co-treatment with LY294002 and bicyclol or PD98059 and bicyclol. The cells were treated as in (A) for 24 h, and the percent of cells in G1 phase was determined by flow cytometry. **c** The bicyclol-mediated protein levels after co-treatment with LY294002 and bicyclol. Cells were pre-treated with both 10 μM LY294002 and 200 μM bicyclol for 6 h. Then, the cells were disrupted, and cellular β-actin, p-AKT (Ser 473), total AKT, p-ERK1/2 (Thr202 and Tyr 204), total ERK, p-Rb (Ser 807) and LC3 I and II were analyzed by western blotting. **d** The bicyclol-mediated protein levels after co-treatment with PD98059 and bicyclol. The cells were pre-treated with 20 μM PD98059 for 30 min and then 200 μM bicyclol was added to the media for 6 h. Then, the cells were disrupted, and cellular β-actin, p-AKT (Ser 473), total AKT, p-ERK1/2 (Thr202 and Tyr 204), total ERK, Ras, p-Rb (Ser 807) and LC3 I and II were analyzed by western blotting. **e** The autophagosomes and autolysosomes were increased by LY294002 and PD98059. The cells were transiently transfected with GFP-RFP-LC3 vectors using Lipofectamine 2000 and incubated for 48 h. Then the cells were treated with both 10 μM LY294002 and 200 μM bicyclol for 24 h, or cells were pre-treated with 20 μM PD98059 for 30 min and then 200 μM bicyclol was added to the media for 24 h. The GFP-RFP-LC3 fluorescence was observed by a confocal microscope, and the number of autophagosomes (*yellow dots*) and autolysosomes (free *red dots*) in each cell were counted by ImageJ. 50 cells for each condition were counted. Bar graphs represent the means ± SD from three independent experiments. (*p < 0.05 versus bicyclol treatment)

To further confirm the central role of the AKT signaling pathway and the Ras/Raf/MEK/ERK signaling pathway in bicyclol-induced cell cycle arrest and autophagy, we co-treated HepG2 cells with bicyclol and LY294002, a PI3K inhibitor, or PD98059, a MEK inhibitor (Fig. 6). The MTT assay results showed that cell proliferation was significantly decreased after 24 h and 48 h of co-treatment with bicyclol and LY294002 compared to treatment with only bicyclol as a control (Fig. 6a). Moreover, cell proliferation was significantly decreased after 48 h of co-treatment with bicyclol and PD98059. Furthermore, the percentage of cells in G1 phase was remarkably increased from 68.7 to 71.8 % after co-treatment with bicyclol and LY294002 (Fig. 6b, and DNA Distribution was presented in Additional file 2B). Additionally, the percentage of cells in G1 phase was increased from 68.7 to 80.9 % after co-treatment with bicyclol and PD98059. The amount of autolysosomes and autophagosomes were also significantly increased after co-treatment with bicyclol and LY294002 compared to treatment with only bicyclol (Fig. 6e). In addition, the LC3-I to LC3-II conversion in AKT-inhibited cells was enhanced. Furthermore, AKT phosphorylation at Ser473 and ERK1/2 phosphorylation at Thr202 and Tyr 204 were inhibited after the co-treatment, which led to Rb dephosphorylation and resulted in an increase in the percent of cells in G1 phase (Fig. 6c). However, the amount of autolysosomes and autophagosomes were significantly increased after treatment with bicyclol and PD98059. The LC3-I to LC3-II conversion in ERK-inhibited cells was enhanced as well (Fig. 6e). In addition, AKT phosphorylation at Ser473 and ERK1/2 phosphorylation at Thr202 and Tyr 204 were inhibited after the co-treatment, which led to Rb dephosphorylation and resulted in an increase in the percent of cells in G1 phase (Fig. 6d, and DNA Distribution was presented in Additional file 2C). Moreover, the expression level of Ras was constant. Taken together, these findings suggested that bicyclol induced cell cycle arrest and autophagy through the PI3K/AKT and the Ras/Raf/MEK/ERK pathways.

### Genetic silencing of AKT and ERK enhanced bicyclol-mediated cell cycle arrest and autophagy

**Fig. 7:**
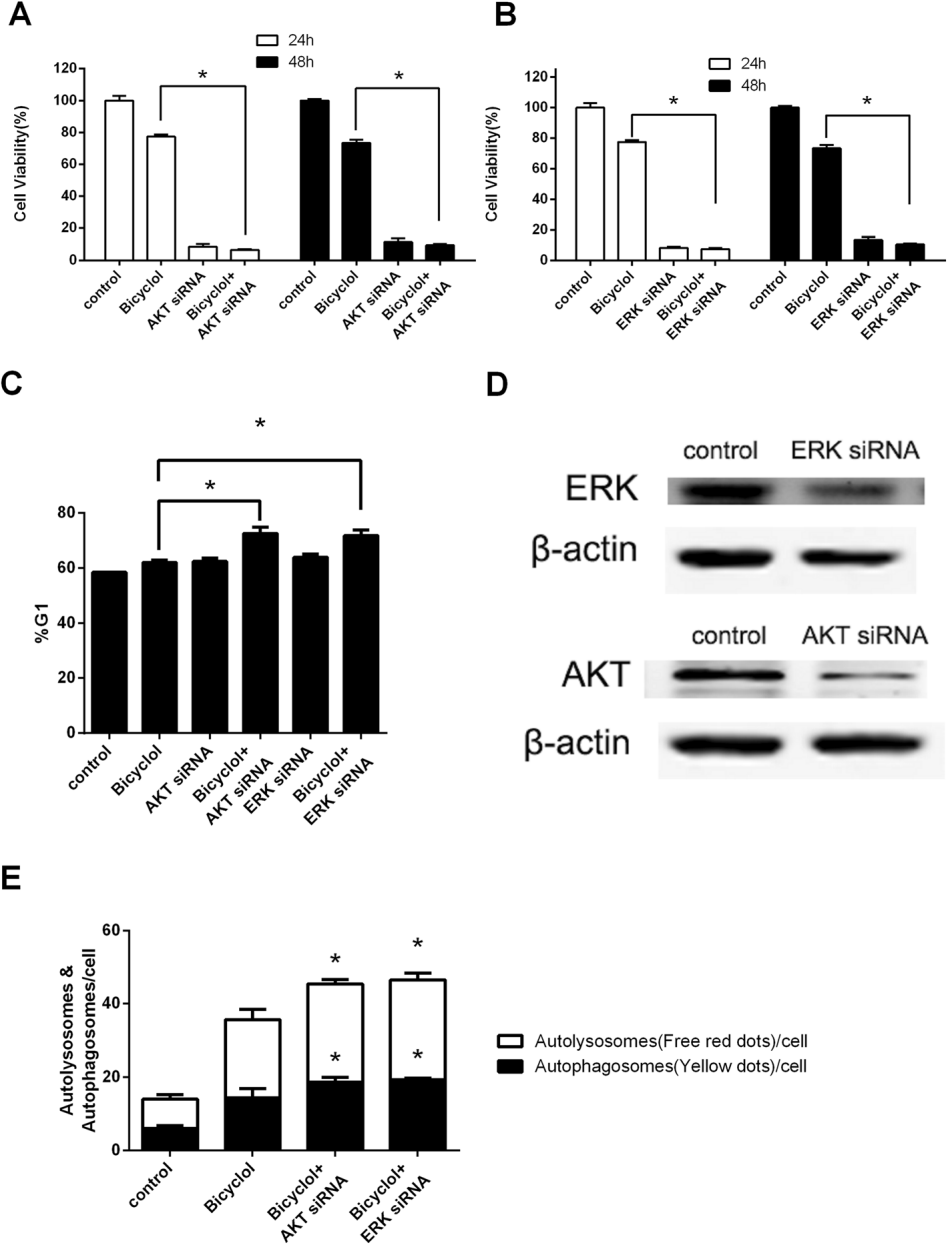
AKT and ERK siRNAs enhanced the anti-proliferative effect of bicyclol in HepG2 cells. **a** Cell proliferation after AKT inhibition and bicyclol treatment. The cells were transiently transfected with the AKT siRNA using Lipofectamine RNAiMAX and incubated for 48 h. Then, the transfected cells were treated with 200 μM bicyclol for 24 or 48 h. The A_570_ was then measured after the MTT incubation. **b** Living cell number after ERK inhibition and bicyclol treatment. The cells were transiently transfected with the ERK siRNA using Lipofectamine RNAiMAX and incubated for 48 h. Then, the transfected cells were treated with 500 μM bicyclol for 24 or 48 h. The A_570_ was then measured after the MTT incubation. **c** The percent of cells in G1 phase after AKT or ERK inhibition and bicyclol treatment. The cells were treated as in (A) with 200 μM bicyclol for 24 h, and the percentage of cells in G1 phase was determined by flow cytometry. **d** The transfection efficiency of the AKT and ERK siRNAs. The cells were transiently transfected with AKT or ERK siRNAs using Lipofectamine RNAiMAX and incubated for 48 h. Then, the cells were disrupted, and cellular β-actin, total AKT and total ERK were analyzed by western blotting. **e** The number of autophagosomes and autolysosomes was increased by AKT or ERK inhibition. The cells were co-transfected with the GFP-RFP-LC3 vector and AKT/ERK siRNAs using Lipofectamine 2000, incubated for 48 h, and then treated with 200 μM bicyclol for another 24 h. The GFP-RFP-LC3 fluorescence was observed by a confocal microscope, and the number of autophagosomes (*yellow dots*) and autolysosomes (free *red dots*) in each cell was counted. Bar graphs represent the means ± SD from three independent experiments. (*p < 0.05 versus bicyclol treatment)

Although LY294002 and PD98059 are relatively selective inhibitors of AKT and MEK, they may influence other proteins that mediate cell cycle or autophagy. Therefore, we also used small interfering RNAs (siRNAs) to specifically silence AKT and ERK and evaluate the effect of AKT and ERK silencing on bicyclol-mediated cell cycle arrest and autophagy. Thus, HepG2 cells were transfected with a pool of siRNAs targeting AKT or ERK before bicyclol treatment. The transfection efficiency was verified by western blot assay (Fig. 7d). As shown in Fig. 7a, the combination of genetic silencing of AKT and treatment with 200 μM bicyclol for 48 h reduced the living cell number to 9.4 %, while treatment with bicyclol alone reduced the living cell number to 73.4 %. Additionally, the combination of genetic silencing of ERK and treatment with 200 μM bicyclol for 48 h reduced the living cell number to 10.6 %, while treatment with bicyclol only reduced the living cell number to 73.4 % (Fig. 7b). The flow cytometry results showed that specific knockdown of AKT and ERK expression enhanced the bicyclol-induced G1 arrest (Fig. 7c, , and DNA Distribution was presented in Additional file 1A). Furthermore, a GFP-RFP-LC3 and siRNA co-transfection was established and investigated by fluorescence microscopy. As shown in Fig. 7e, genetic silencing of AKT and ERK significantly enhanced the bicyclol-induced increase in the amount of autolysosomes and autophagosomes compared with bicyclol treatment alone. These influences of siRNA-mediated AKT and ERK silencing on bicyclol-induced cell cycle arrest and autophagy agree with the results from the chemical inhibitors, indicating that bicyclol suppresses the PI3K/AKT and the Ras/Raf/MEK/ERK pathways, leading to LC3 conversion, inhibition of the growth regulatory signals of G1 phase, and eventually cell cycle arrest at G1 phase and autophagy.

## Discussion

At present, bicyclol is a novel synthetic drug that has been widely used in the clinic to treat patients with chronic hepatitis B viral infections [6]. The previous studies focused on its protective effects against hepatotoxin-induced liver injury, but the anti-cancer potential of bicyclol remained unexplored. From previous studies [13], bicyclol has protective effects and induces expression of heat shock protein 27 under 100uM at less than 24 h. In this study, we found that bicyclol induces cell cycle arrest at G1 phase and autophagy at more than 100uM, and we also identified the molecular mechanism, showing that bicyclol suppresses both the PI3K/AKT pathway and the Ras/Raf/MEK/ERK pathway and downregulates cyclin D, cyclin E and mTOR, leading to Rb dephosphorylation and the conversion of LC3I to LC3II.

We found that bicyclol treatment of HepG2 cells caused a dose-dependent increase in the percentage of cells in G1 phase. Therefore, we specifically focused on proteins that regulate the cell cycle. CDK2 and CDK4 play a central role in cell cycle progression by forming complexes with cyclin E and D1, respectively [30, 31]. By binding to cyclin Ds, CDK4 phosphorylates the Rb protein to release Rb from the E2F complex, leading to transactivation of the E2F target genes important for S phase [32–34]. By binding to cyclin Es, CDK2 also can phosphorylate the Rb protein and control the transition into S phase [35]. As shown in Fig. 3, bicyclol not only inhibited CDK2-cyclin E and CDK4-cyclin D expression but also suppressed Rb phosphorylation in a dose-dependent manner. Intrinsic CDK inhibitors, such as p21^CIP1^ and p27^KIP1^, which appear to be the primary negative regulators during proliferation in a variety of cell types, induce G1 cell cycle arrest by binding to the CDK–cyclin complex and inhibiting its kinase activity [36–38]. Our results suggested that p21^CIP1^ and p27^KIP1^ upregulation, inhibition of levels of the CDK2-cyclin E and CDK4-cyclin D complexes, and Rb down-regulation contribute to the anti-proliferative effects of bicyclol in HepG2 cells.

We investigated the pathways that regulate cell cycle arrest at G1/S checkpoint to further confirm which pathways were downstream of bicyclol. In this study, bicyclol dephosphorylates Akt at Ser 473 and downregulates the PI3K/AKT pathway in HepG2 cells. The PI3K/AKT pathway plays a major role in many carcinogenic processes, such as cell growth and differentiation, and AKT phosphorylation at Ser473 is essential for maximal Akt activation [39]. Compelling evidence suggests that expression of phosphor-Ser473 Akt in primary human breast cancers was statistically correlated with p27^KIP1^ expression in the tumor cytosol [40]. Moreover, activated Akt directly phosphorylates and inhibits p21^CIP1^ [41], indirectly activates cyclin D, and, thus, induces cell cycle progression [42]. In this study, we speculated that the bicyclol-induced Akt inactivation subsequently up-regulated the level of p21^CIP1^ and p27^KIP1^ transcription, thereby blocking cell cycle progression at G1 phase by suppressing CDK2 and CDK4 activity. The Ras/Raf/MEK/ERK pathway is also inhibited by bicyclol. Blocking ERK activity blocks cyclin D1 expression and cell proliferation [43]. Additionally, activated ERK is essential for the assembly of the cyclin E/CDK2 complex [44]. Thus, ERK plays an important role in the G1/S checkpoint. In this study, we suggested that the bicyclol-induced ERK inactivation downregulated the cyclin D/CDK4 and cyclin E/CDK2 complexes. Furthermore, the PI3K/AKT and the Ras/Raf/MEK/ERK pathways can interact in multiple ways. MEK can activate Akt activity in hematopoietic cells [45], and Akt can contribute to Raf-1 inactivation in some cells. However, it is not clear how bicyclol mediates the two pathways, which needs further studies.

The PI3K/AKT and the Ras/Raf/MEK/ERK pathways also play an important role in autophagy. Activated Akt directly phosphorylates the protein encoded by the TSC2 tumor suppressor gene [46]. The phosphorylation blocks TSC2 binding with TSC1, and subsequently prevents formation of the TSC1/2 complex [47]. Akt can also inhibit PRAS40, which then activates mTORC1 and induces autophagy [29]. ERK also phosphorylates and inhibits TSC1/TSC2, which then activates mTORC1 and induces autophagy [48]. The mTOR complex 1 (mTORC1) consists of mTOR, RAPTOR, PRAS40, mLST8, DEPTOR, and the Tti1/Tel2 complex [49–52]. mTORC1 can phosphorylate the autophagy-related gene 13 (ATG13) [53] and the autophagy/beclin 1 regulator 1 (AMBRA1) [54], which inhibits the autophagy-initiating UNC-5-like autophagy activating kinase (ULK) complex. On the other hand, the mTORC1 can also regulate the VPS34 complex by phosphorylating ATG14 L [55]. Therefore, mTORC1 inhibition induces ULK1/2 complex and the VPS34 complex activity, leading to the conversion of LC3I to LC3II, a specific marker of autophagosome formation, and eventually induces autophagy. Our findings demonstrated that bicyclol inhibited p-mTOR, converted LC3I to LC3II, and induced autophagosome formation and autophagy. The amount of autolysosomes and autophagosomes were significantly increased after co-treatment with bicyclol and LY294002 (or PD98059). In addition, AKT or ERK knockdown by siRNA enhanced bicyclol-induced autophagy. Based on the above analysis, we conclude that the bicyclol-induced autophagy is closely associated with the PI3K/AKT and the Ras/Raf/MEK/ERK pathways.

## Conclusions

In conclusion, bicyclol inhibited cell cycle progression at G1 phase and induced autophagy in HepG2 cells, and the drug didn’t increase cell apoptosis or necrosis. The anti-proliferative effect of bicyclol was considered as the result of combination of cell cycle arrest and autophagy, leading to induced cell proliferation in MTT results. Our mechanistic study indicated that the cytotoxicity of bicyclol is closely associated with the inhibition of the PI3K/AKT pathway and the Ras/Raf/MEK/ERK pathway. The results contribute to our understanding of bicyclol and provide clear evidence for its promising potential in preclinical and clinical situations.

## Additional files

Additional file 1: (A) The DNA distribution of cells treated with bicyclol-siRNA-, bicyclol + siRNA-,bicyclol-AKT siRNA+, bicyclol + AKT siRNA+, bicyclol-ERK siRNA+ and bicyclol + ERK siRNA+. The siRNA was transfected as mentioned in Methods. Then the cells were treated with bicyclol for 24 h. (B) MTT results of HepG2 cell viability with treatment 500 μM Bicyclol or 10 μM Sorafenib. (TIF 696 kb) Additional file 2: (A) The DNA distribution of cells treated with bicyclol-AKT cDNA-, bicyclol-AKT cDNA+, bicyclol + AKT cDNA- and bicyclol + AKT cDNA+. The cDNA was transfected as mentioned in Methods. Then the cells were treated with bicyclol for 24 h (B) The DNA distribution of cells treated with bicyclol or/and LY294002 for 24 h. (C) The DNA distribution of cells treated with bicyclol or/and PD98059 for 24 h. (TIF 809 kb)

## Abbreviations

3-MA: 3-methyladenine
AKT: v-akt murine thymoma viral oncogene homolog
CDK: Cyclin-dependent kinases
ERK: Extracellular signal-regulated kinases
FITC: Fluorescein isothiocyanate
GFP: Green fluorescent protein
LC3: Light chain 3
Mdm2: Mouse double minute 2 homolog
MEK: Mitogen/extracellular signal-regulated kinase
mTOR: Mammalian target of rapamycin
mTORC1: mTOR complex 1
NF-kB: Nuclear factor kappa-light-chain-enhancer of activated B cells
PAGE: Polyacrylamide gel electrophoresis
PBS: Phosphate buffer saline
PI: Propidium iodide
PI3K: Phosphatidylinositide 3-kinases
Rb: Retinoblastoma protein
RFP: Red fluorescent protein
siRNA: Small interfering RNA
TSC: Tuberous sclerosis complex
