# Supplementary figures and images for: Bicyclol induces cell cycle arrest and autophagy in HepG2 human hepatocellular carcinoma cells through the PI3K/AKT and Ras/Raf/MEK/ERK pathways
Source: BMC Cancer. 2016 Sep 21;16:742. doi: 10.1186/s12885-016-2767-2 (PMC5031284; doi:10.1186/s12885-016-2767-2)

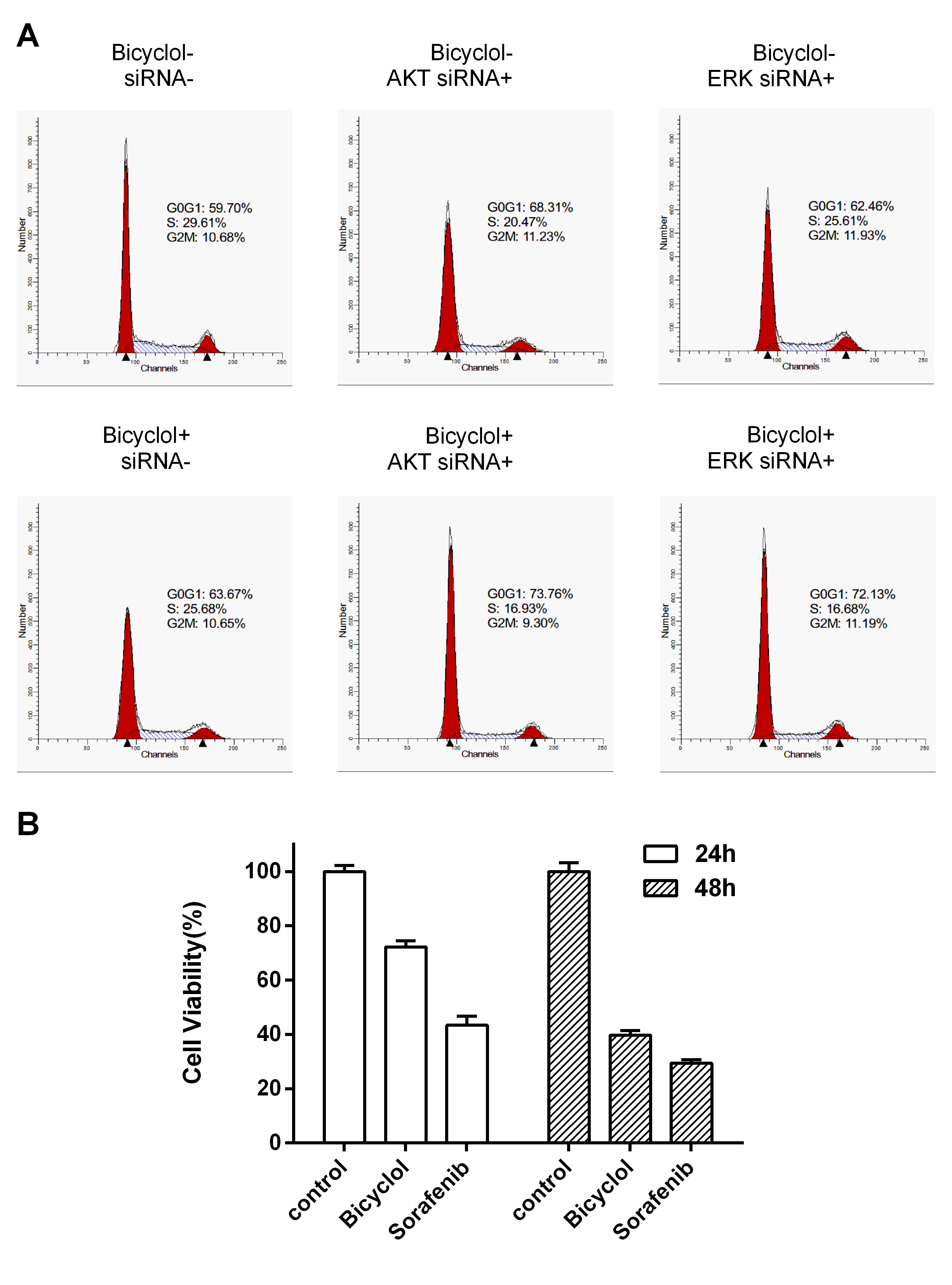

Supplement: Additional file 1: — (A) The DNA distribution of cells treated with bicyclol-siRNA-, bicyclol + siRNA-,bicyclol-AKT siRNA+, bicyclol + AKT siRNA+, bicyclol-ERK siRNA+ and bicyclol + ERK siRNA+. The siRNA was transfected as mentioned in Methods. Then the cells were treated with bicyclol for 24 h. (B) MTT results of HepG2 cell viability with treatment 500 μM Bicyclol or 10 μM Sorafenib. (TIF 696 kb) [file 12885_2016_2767_MOESM1_ESM.tif]

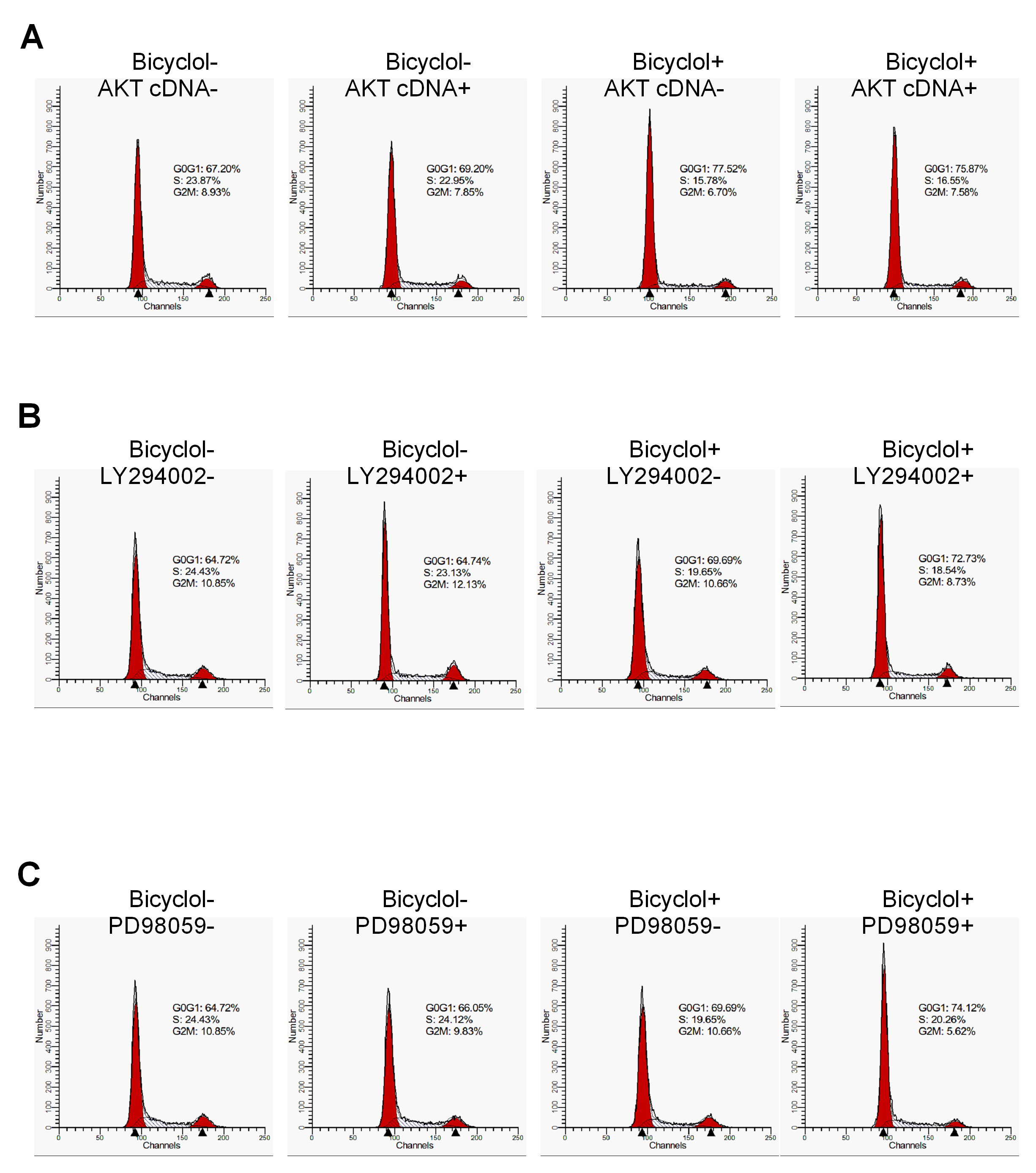

Supplement: Additional file 2: — (A) The DNA distribution of cells treated with bicyclol-AKT cDNA-, bicyclol-AKT cDNA+, bicyclol + AKT cDNA- and bicyclol + AKT cDNA+. The cDNA was transfected as mentioned in Methods. Then the cells were treated with bicyclol for 24 h (B) The DNA distribution of cells treated with bicyclol or/and LY294002 for 24 h. (C) The DNA distribution of cells treated with bicyclol or/and PD98059 for 24 h. (TIF 809 kb) [file 12885_2016_2767_MOESM2_ESM.tif]
